# Transcriptome-wide expression profiling of *Sporothrix schenckii* yeast and mycelial forms and the establishment of the Sporothrix Genome DataBase

**DOI:** 10.1099/mgen.0.000445

**Published:** 2020-10-09

**Authors:** Domenico Giosa, Maria Rosa Felice, Letterio Giuffrè, Riccardo Aiese Cigliano, Andreu Paytuví-Gallart, Carla Lo Passo, Cinzia Barresi, Enrico D'Alessandro, Huaiqiu Huang, Giuseppe Criseo, Héctor M. Mora-Montes, Sybren de Hoog, Orazio Romeo

**Affiliations:** ^1^​ Department of Clinical and Experimental Medicine, University Hospital of Messina, Messina 98125, Italy; ^2^​ Department of Chemical, Biological, Pharmaceutical and Environmental Sciences, University of Messina, Messina 98166, Italy; ^3^​ Department of Veterinary Sciences, Division of Animal Production, University of Messina, Messina 98168, Italy; ^4^​ Sequentia Biotech SL, Barcelona 08005, Spain; ^5^​ Department of Dermatology and Venereology, Third Affiliated Hospital of Sun Yat-sen University, Guangzhou 510630, Guangdong, PR China; ^6^​ Department of Dermatology and Venereology, Baoan District People’s Hospital of Shenzhen, Shenzhen 518012, PR China; ^7^​ Departamento de Biología, División de Ciencias Naturales y Exactas, Campus Guanajuato, Universidad de Guanajuato, Guanajuato 36050, Mexico; ^8^​ Center of Expertise in Mycology, Radboud University Medical Center/Canisius Wilhelmina Hospital, Nijmegen, The Netherlands

**Keywords:** *Sporothrix schenckii*, sporotrichosis, gene expression, RNA-seq, overlapping genes, long non-coding RNAs

## Abstract

*Sporothrix schenckii* is a dimorphic fungus existing as mould in the environment and as yeast in the host. The morphological shift between mycelial/yeast phases is crucial for its virulence, but the transcriptional networks implicated in dimorphic transition are still not fully understood. Here, we report the global transcriptomic differences occurring between mould and yeast phases of *S. schenckii*, including changes in gene expression profiles associated with these distinct cellular phenotypes. Moreover, we also propose a new genome annotation, which reveals a more complex transcriptional architecture than previously assumed. Using RNA-seq, we identified a total of 17 307 genes, of which 11 217 were classified as protein-encoding genes, whereas 6090 were designated as non-coding RNAs (ncRNAs). Approximately ~71 % of all annotated genes were found to overlap and the different-strand overlapping type was the most common. Gene expression analysis revealed that 8795 genes were differentially regulated among yeast and mould forms. Differential gene expression was also observed for antisense ncRNAs overlapping neighbouring protein-encoding genes. The release of transcriptome-wide data and the establishment of the Sporothrix Genome DataBase (http://sporothrixgenomedatabase.unime.it) represent an important milestone for *Sporothrix* research, because they provide a strong basis for future studies on the molecular pathways involved in numerous biological processes.

## Data Summary

- The data supporting the conclusions of this article have been deposited in the National Center for Biotechnology Information (NCBI) Gene Expression Omnibus (GEO) database and are accessible through GEO series accession number GSE145856 (www.ncbi.nlm.nih.gov/geo/query/acc.cgi?acc=GSE145856). The Illumina raw reads have also been submitted into the NCBI Sequence Read Archive (SRA) database under the following study accession number SRP194160 (www.ncbi.nlm.nih.gov/sra/?term=SRP194160) associated with BioProject ID PRJNA539953.
- All supplementary material (Supplementary File S1 and Tables S1–S6) has been deposited in Figshare and is available within the project ‘Sporothrix schenckii RNA-Seq’ at https://doi.org/10.6084/m9.figshare.12894761.v1.
- All genomic data, including gene models and expression data, can also be viewed, inspected and/or downloaded from the Sporothrix Genome DataBase (http://sporothrixgenomedatabase.unime.it).

**Impact Statement**

The genetic knowledge of *Sporothrix schenckii* is, at present, very poor, and even less is known about the transcriptional organization and regulation of the genes that control the myriad of biological processes, including dimorphism and the associated saprophytic/pathogenic change, that make this fungus a successful pathogen of humans and animals. Our results expand current knowledge on the content, organization, structure and expression of *S. schenckii* genes by providing, for what is believed to be the first time, fundamental information on the transcriptional activity of its non-coding genome.

## Introduction

*Sporothrix* is a fungal genus comprising over 50 species [1, 2] that are largely known as environmental saprophytes, which can be isolated from soil, plants and even animals [3]. A few species are also able to infect humans and other mammals, causing a sub-acute or chronic disease called sporotrichosis [2, 4].

In a recent taxonomic revision of the genus *Sporothrix* [1], the disease-causing species have been grouped in the so-called ‘pathogenic clade’, which contains the four human pathogens (*Sporothrix schenckii*, *Sporothrix brasiliensis*, *Sporothrix globosa* and *Sporothrix luriei*) responsible for almost all cases of sporotrichosis occurring worldwide [2, 5]. One of the most striking phenotypic traits of these species is their ability to switch reversibly between two different morphologies in response to temperature shifts [6]. In the environment, or at 25 °C, they grow as moulds, forming conidia, whereas at 37 °C, or when acquired by humans, they are capable of converting into pathogenic yeasts that effectively overcome the host immune defences and cause disease [7]. Surprisingly, despite their very close phylogenetic relationships, the members of the pathogenic clade showed remarkable differences in many aspects of their basic biology, including epidemiology, antifungal resistance, virulence and pathogenicity [8–12]. Nevertheless, the study of pathogenic *Sporothrix* species is still mainly limited to their clinical aspects, laboratory diagnosis and therapy [6], and little progress has been made in understanding the genetics and gene regulatory networks that control many biological processes, including dimorphism and the associated saprophytic/pathogenic change.

In this study, we report, for what is believed to be the first time, a comprehensive transcriptomic analysis of *S. schenckii* in relation to two different cellular morphologies (yeast-form and hyphal-form) obtained by growing the fungus at 37 and 25 °C, respectively. Moreover, we also present the Sporothrix Genome DataBase (SGDB; http://sporothrixgenomedatabase.unime.it), a web-based genomic platform for integrating RNA-seq expression data and exploring gene models in the *S. schenckii* genome.

## Methods

### Fungal strain and culture conditions

In this study, we examined the reference strain *S. schenckii* ATCC MYA-4821 (formerly referred to as strain 1099–18), as its genome was already sequenced and *in silico* annotated in 2014 (GenBank assembly accession number GCA_000961545.1) [13]. To induce the formation of the yeast-like and mould phases, as well as the expression of genes associated with these two different morphologies, six test tubes, each containing 25 ml YPD broth (1 % yeast extract, 2 % peptone and 2 % dextrose), were inoculated with 50 µl of a standardized suspension of *S. schenckii* conidia from a 4-day-old YPD agar culture grown at 28 °C. Conidia were harvested by washing the agar plate with PBS 1x, pH 7.4, and removed from the mycelium with a sterile glass rod. Then, the conidial suspension was ﬁltered through sterile glass wool to remove mycelial fragments and standardized to McFarland’s number 1 (~10^7^ cells ml^−1^) using a Den-1B benchtop densitometer (bioSan). Inoculated YPD broth tubes were incubated in triplicate at 37 and 25°C, and monitored daily up to 6 days to confirm the expected morphology of the organism (yeast-like cells at 37 °C and hyphae at 25 °C).

### RNA extraction, Illumina TruSeq stranded library preparation and sequencing

Total RNA extraction was performed by using a RiboPure yeast extraction kit (Thermo Fisher Scientific), following the manufacturer’s instructions. Purified RNA was quantified spectrophotometrically at 260 and 280 nm, and the RNA integrity was evaluated with an Agilent 2100 Bioanalyzer instrument using an RNA 6000 Nano kit (Agilent Technologies). High-quality total RNA (OD_260/280_ ≥2.0; RNA Integrity Number, RIN value ≥8.0) was sent to IGATech (https://igatechnology.com) for library preparation using TruSeq stranded mRNA chemistry (Illumina) and paired-end (2×150 bp) sequencing on the Illumina NextSeq 500 platform. A total of three biological replicates were sequenced for each culture condition tested.

### Transcriptome assembly and genome annotation

For each sequenced library**,** Illumina raw reads were pre-processed with the program Trimmomatic (v.0.39) [14] to remove adapters and sequences with low Phred-scores (minimum Phred-quality score ≥25; minimum length 35 bp), and then used for downstream bioinformatic analyses including genome annotation and differential gene expression analysis. The software star (v.2.7.1a) [15] was used to map cleaned reads on the previously published and annotated *S. schenckii* reference genome (GenBank accession no. GCA_000961545.1). Mapping quality was evaluated using Qualimap 2 software (v.2.2.1) [16].

Although over 99 % of the reads mapped uniquely to the reference genome, a significant fraction of these (>30 %) were located in unannotated genomic regions. Therefore, before gene expression analysis, we decided to re-annotate the existing *S. schenckii* ATCC MYA-4821 genome using our RNA-seq data. Genome annotation was performed using the software maker (v.3.00.0) [17] and a combination of other resources, including snap (v.2.39) [18] and augustus (v. 3.3.1) [19] *ab initio* gene predictors. In addition, a genome-guided transcriptome was generated with Trinity software (v.2.5.1) [20] using our RNA-seq data (minimum coverage 10 reads).

To obtain the first draft of the annotation, in the first round of the maker pipeline we provided the original *S. schenckii* genome annotation (GCF_000961545.1_S_schenckii_v1_genomic.gff), the whole transcriptome created by Trinity and two whole data sets of *Ophiostomatales* [National Center for Biotechnology Information (NCBI) txid5151] expressed sequence tags (ESTs), and protein sequences downloaded from the NCBI Protein and Nucleotide databases (www.ncbi.nlm.nih.gov). However, before using these reference data sets in the annotation pipeline, the sequence redundancy was removed by cd-hit software v.4.8.1 [21] using a sequence similarity threshold of 90 %. Subsequently, to improve the accuracy of the *S. schenckii* gene models, we performed four iterations of training and prediction in order to generate hidden Markov models (HMMs) for training *ab initio* gene-finding software (first round with snap, then augustus) included in the maker pipeline.

Since many transcripts were not automatically annotated by maker, we manually extracted all transcript evidence reported by maker as ‘match’. GenomeTools (v.1.5.10) [22] was first used to properly format the annotation, and then to extract transcripts and corresponding proteins. Transcripts were aligned against the UniProt database (www.uniprot.org; release 2019_03) using the blast algorithm (v.2.8.1) [23]. Transcripts without any match in the UniProt database were further investigated by using InterProScan (v. 5.35–74.0) [24], Coding Potential Calculator 2 (cpc2) [25] and Infernal (v.1.1.2) [26] programs in order to evaluate the presence of protein domains, non-coding and potential coding sequences. However, transcripts without matched known protein-encoding functions were predicted as novel, potential, long non-coding RNA (lncRNA) genes based on the following criteria: (i) the assembled transcripts showed definite strand information; (ii) their length was ≥200bp; and (iii) their coding potential was predicted by the cpc2 program using specific sequence intrinsic features: ORF, length, ORF integrity and isoelectric point [25]. Only transcripts labelled as ‘noncoding’ in the cpc2 output were kept.

RepeatMasker (v.4.0.9) (http://www.repeatmasker.org) and Tandem Repeat Finder (v.4.09) (https://tandem.bu.edu/trf/trf.html) software were used to predict repetitive and transposable elements. The tRNAs were detected using tRNAscan-SE software (v.1.3.1) [27]. All the proteins were functionally annotated with pannzer2 software [28].

### Identification of cis-sense–antisense (cis-SAS) gene pairs, same-strand overlapping genes and overlapping gene groups

**Fig. 1.**
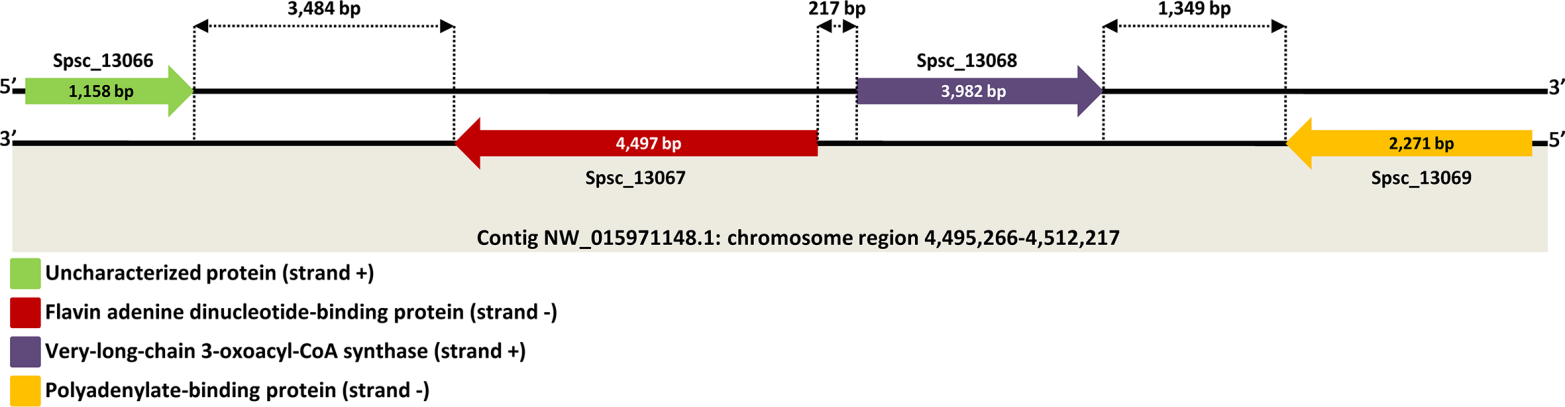
A representative genomic region showing four different protein-encoding genes transcribed from opposite DNA strands. The image was built based on the information provided by the strand-specific RNA-seq data obtained in this study.

**Fig. 2.**
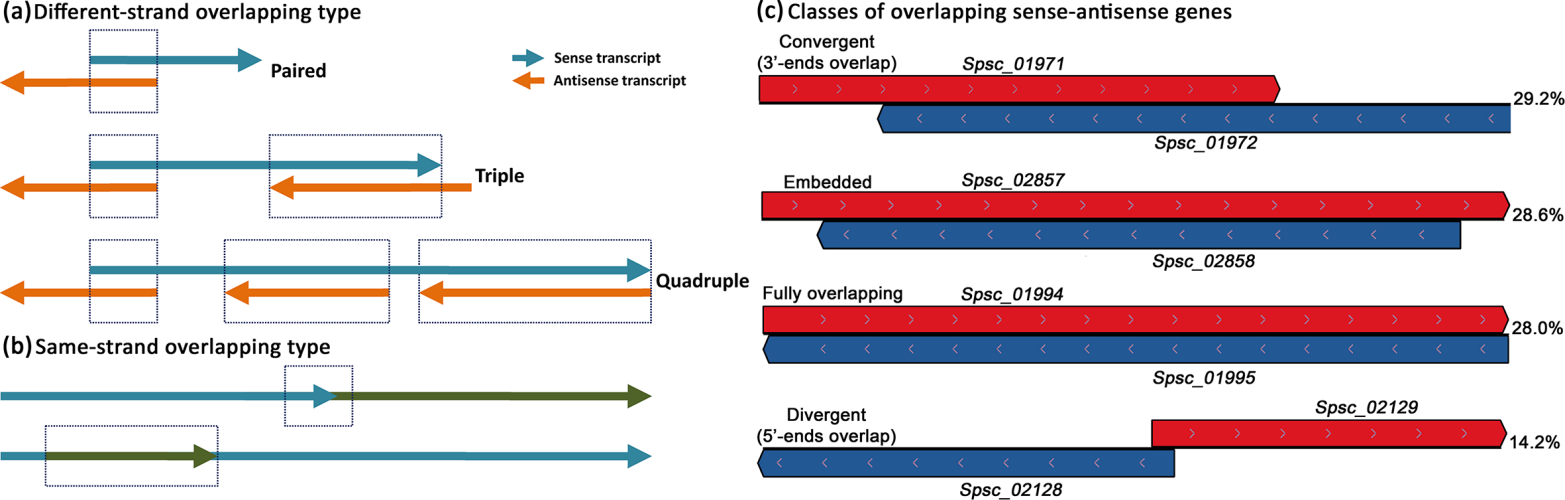
Different types of overlapping genes. (a) Some examples of different-strand overlapping genes in which two or more genes are transcribed from opposite DNA strands. Gene groups (paired, triple, quadruple) were established based on the number of sense–antisense transcripts involved in each single uninterrupted genomic region. (b) Same-strand overlapping genes in which two adjacent genes overlap partially (or entirely) with each other by sharing a common genomic region. (c) Schematic representation of paired cis-SAS genes according to the orientation of the overlapping transcripts and their transcriptional direction. The percentage of each configuration found in the *S. schenckii* genome is shown on the right.

Stranded RNA-seq data obtained in this study provided potentially valuable information to map sense and antisense transcripts, and thereby accurately predict genomic regions containing genes arranged on opposite DNA strands (Fig. 1). We defined overlapping sense–antisense regions as those genomic traits in which one sense transcript overlaps (at least 1 nucleotide) with an antisense transcript (Fig. 2). However, it should be pointed out that a single sense transcript could overlap with two or more different antisense transcripts by generating multiple overlapping areas (Fig. 2a). Nevertheless, each overlap site within these composite gene regions was counted once. According to this criterion, we searched the *S. schenckii* genome for overlapping regions by using the BEDtools intersect utility (v2.28.0) [29] and custom bash scripts. Subsequently, we counted the number of cis-SAS genes occurring in these regions and classified them into different groups (paired, triple, quadruple, quintuple and so on), based on the number of sense–antisense transcripts involved in each single uninterrupted genomic region (Fig. 2a). However, in addition to the type of overlapping transcripts described above, generically known as different-strand overlapping type, there is also another type of overlapping genes called same-strand overlapping type, in which the genes involved are transcribed from the same DNA strand [30] (Fig. 2b). Therefore, we also searched for same-strand overlapping genes by using BEDtools [29], and the boundaries of newly annotated genes defined by the start and end gene positions reported on assembled genome scaffolds. Genes were considered to overlap with each other if they shared at least 1 nucleotide.

### Differential gene expression and gene ontology (GO) enrichment analyses

Using our new comprehensive genome annotation, we carried out a global gene expression analysis for identifying genes specifically expressed during yeast-like growth at 37 °C and/or in mould-producing cultures at 25 °C. The high-quality reads of each sample were mapped onto the newly annotated reference genome with the star program (v.2.7.1a) [15] and the quality of mapping was assessed using Qualimap 2 software (v.2.2.1) [16]. The number of RNA-seq reads or read-pairs overlapping the genomic features was counted by featureCounts software integrated into the Subreads package (v.1.6.4) [31]. For each RNA-seq library, fragments per kilobase of transcript per million mapped reads (FPKM) values were calculated and used to estimate the number of transcripts detected. According to another study [32], a FPKM cut-off >1 in at least one of the six libraries was used to establish whether a transcript was expressed by *S. schenckii* cells from which RNA was extracted. The transcripts whose FPKM value was below the threshold value of 1 were considered not detected by the RNA-seq method employed.

The normalization of raw counts was performed using the trimmed means of M-values (TMM) method in the HTSFilter package [33]. Differentially expressed genes (DE genes) were identified using the edgeR package [34]. The genes whose expression levels showed an absolute log_2_ fold-change (log_2_FC) value ≥1 and a false discovery rate (FDR) ≤0.05 were considered to be significantly differentially expressed. GO enrichment analysis was performed on the DE genes using in-house scripts following the methods outlined in agriGO (v.2.0) [35].

### Quantitative real-time PCR (qRT-PCR)

RNA-seq data were validated by qRT-PCR using a panel of randomly selected coding- and non-coding transcripts (Table S1) representing DE genes and non-differentially expressed genes for the two conditions tested. PCR primers were designed and validated using Vector NTI software (version 10.3.0; Invitrogen). For the quantification of transcripts encoded by overlapping loci, primers were designed in non-overlapping regions of the gene to avoid the amplification and quantitation of the corresponding complementary RNA sequence.

Total RNA (2 µg) was digested with DNase I (Sigma-Aldrich), following manufacturer’s instructions, and retrotranscribed by a RevertAid first strand cDNA synthesis kit (Thermo Fisher Scientific) using oligo(dT) at 42 °C for 1 h, followed by a reverse transcriptase denaturation step at 70 °C for 10 min. qRT-PCR was performed using the StepOnePlus real-time PCR system (Applied Biosystem) with PowerUp SYBR Green master mix (Thermo Fisher Scientific) and the primer sequences listed in Table S1.

Relative gene expression quantification was calculated by the 2^−ΔΔ^ *^C^*_^t^_method [36] using the β-tubulin gene as an endogenous housekeeping gene. Spearman’s correlation coefficient (r_s_) was used to estimate the degree of correlation between gene expression levels measured by qRT-PCR and RNA-seq methods.

### SGDB

In order to make our data easily accessible and available to the scientific community, we developed the SGDB (http://sporothrixgenomedatabase.unime.it), an online resource that offers an integrated toolset for accessing, analysing and exploring genomics data. Currently, SGDB provides gene expression data as well as protein and genomic sequences for *S. schenckii* only, but it could be integrated in the future with additional genomic/transcriptomic data from other *Sporothrix* species.

The web server was built using Node JS (version 8.9.4) with the Express framework (version 4.16) in the back-end. Data are stored in files read by the back-end and supplied to the front-end, which consists of mainly html/css and JavaScript, in the corresponding format (e.g. tables or images). A JavaScript-based genome browser (JBrowse v.1.12.3) [37] was also integrated to allow users to quickly view large-scale RNA-seq data and gene annotations along the reference genome in an interactive manner. The SGDB also incorporates the blast search tool allowing investigators to query any sequence against the genome, transcriptome or proteome of *S. schenckii* using either blastn or blastp algorithms.

## Results

### RNA sequencing, transcriptome statistics and genome annotation

Illumina whole-transcriptome shotgun sequencing produced a total of 98 949 246 reads of which 98 837 150 (~99.9 %) were high-quality reads (clean reads) used for further genomics analysis (Table 1). The total size of the sequenced reads was 245.92 Gbp. The overall transcriptome statistics of the six processed biological samples are shown in Table 1.

**Table 1.** Statistics of the RNA-seq data generated in this study

Sequence characteristic	Mould-form			Yeast-form			Total
SSC_1	SSC_2	SSC_3	SSC_4	SSC_5	SSC_6		
Raw reads	15 840 139	15 759 028	13 714 380	19 217 684	18 303 397	16 114 618	98 949 246
Cleaned reads	15 821 431	15 743 831	13 692 566	19 196 851	18 283 034	16 099 437	98 837 150
Read 1 (unpaired)	8668	6371	9843	8277	7517	6166	46 842
Read 2 (unpaired)	3620	3382	4401	4124	4722	2917	23 166
Total mapped*	15 766 109	15 688 507	13 619 058	19 156 060	18 246 967	15 831 742	98 308 443
Uniquely assigned†	12 095 953	12 325 194	10 882 445	16 019 278	14 055 739	13 021 760	78 400 369
Multiply mapped‡	482 067	483 333	432 399	802 480	715 040	692 013	3 607 332
No features mapped§	3 188 089	2 879 980	2 304 214	2 334 302	3 476 188	2 117 969	16 300 742
G+C content (mol%)	50.70	51.35	52.34	54.68	54.46	56.19	53.28||

*Total number of reads aligned on the *S. schenckii* reference genome (v2 annotation).

†Total number of reads mapped to unique features in the newly annotated *S. schenckii* genome (v2 annotation).

‡Total number of reads mapped to multiple locations in the *S. schenckii* genome (v2 annotation).

§Total number of reads mapped to unannotated (v2 annotation) genomic regions.

||Mean value.

The first level of difference between the biological replicates of the two conditions examined was that the data set of the RNA-seq reads produced by the mycelia showed a lower mean G+C content (~51.5 mol%) than that associated with the yeast-form (mean G+C content 55.1 mol%) (Student-*t P* value=0.003634) (Table 1). The raw RNA-seq reads have been deposited in the NCBI Sequence Read Archive (SRA) database and are available under BioProject ID PRJNA539953.

Using our RNA-seq data, we were able to refine the initial set of *S. schenckii* ATCC MYA-4821 gene models (referred to hereafter as the v1 annotation) that was originally generated using only computational methods and, therefore, consisted solely of *ab initio* gene predictions (10 293 protein-encoding genes and 139 tRNAs) [13] (Table 2). Our genome-wide reannotation (v2 annotation) provides an extended and improved annotation of the *S. schenckii* genome by unravelling unreported aspects of its transcriptional architecture (Table 2). Overall, 17 307 genes were identified by integrating our RNA-seq data into the genome annotation pipeline. Most of them (64.8 %; 11 217) showed a hit on at least one of the four databases inspected and were classified as protein-encoding genes, whereas 5929 (34.2 %) lacked evidence for a canonical or functional ORF and were predicted as potential lncRNAs. Moreover, a total of 24 rRNAs, 134 tRNAs and 3 snRNAs (small nuclear RNAs) were also identified (Table 2). The updated v2 annotation of the *S. schenckii* ATCC MYA-4821 genome is available as supplementary material (Supplementary File S1) in the form of a GFF file or can be downloaded from the SGDB (http://sporothrixgenomedatabase.unime.it).

**Table 2.** Comparison of genome-wide statistics for the v1 and v2 annotations of the *S. schenckii* ATCC MYA-4821 genome

Sequence characteristic	Annotation version	
	v1	v2
Total genes	10 432	17 307
Protein-encoding genes	10 293	11 217
Gene length		
Mean (bp)	~1612	~2101
Total (bp)	16 590 731	21 625 318
Gene length/genome (%)	51.23	66.78
Total exons	20 756	30 392
Exon length		
Mean (bp)	~757	~1132
Total (bp)	7 796 060	11 657 420
Exon length/genome (%)	24.07	36.00
lncRNAs	na	5929
rRNA loci	na	24
tRNA loci	139	134
snRNA loci	na	3

na, Not available.

**Fig. 3.**
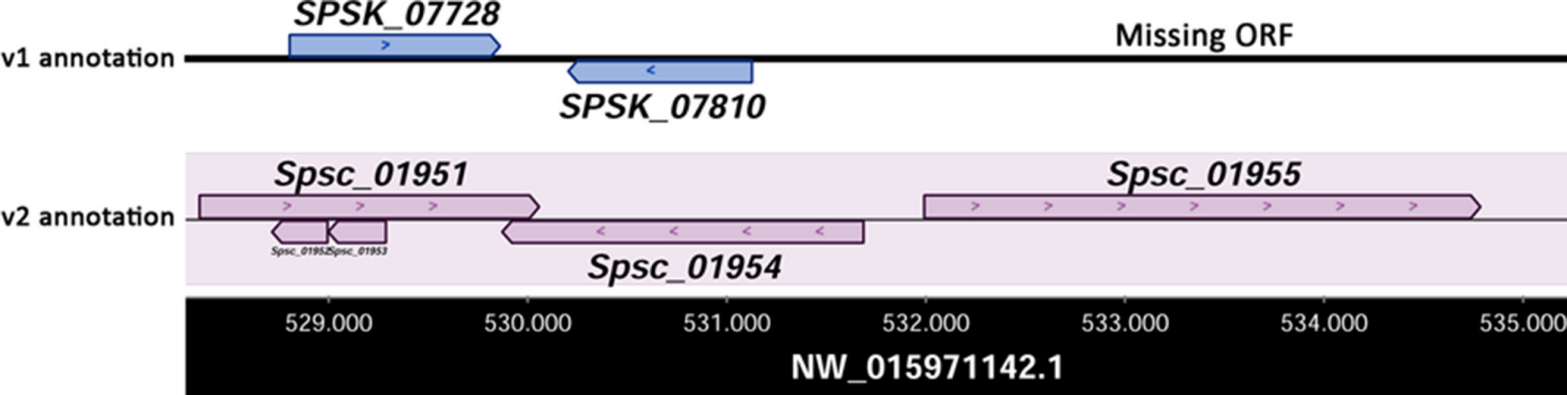
Schematic comparison of v1 and v2 genome annotations illustrating the discovery of additional gene models and the improvement of gene model boundaries.

According to our new genome annotation, we confirmed 98.9 % (10 180/10 293) of the previously reported protein-encoding genes [13] and identified 132 novel protein-encoding sequences within genomic regions that were not annotated in the previous published v1 version (GenBank accession no. GCA_000961545.1) (Table S1). An example is presented for the ORF Spsc_01955, reported in Fig. 3, encoding a heat-shock protein, Hsp98. Table 2 shows the comparison of genome-wide statistics for the v1 and v2 annotations of the *S. schenckii* ATCC MYA-4821 genome.

### Strand-specific RNA-seq reveals a large number of antisense RNAs (asRNAs) overlapping protein-encoding and non-coding genes

**Fig. 4.**
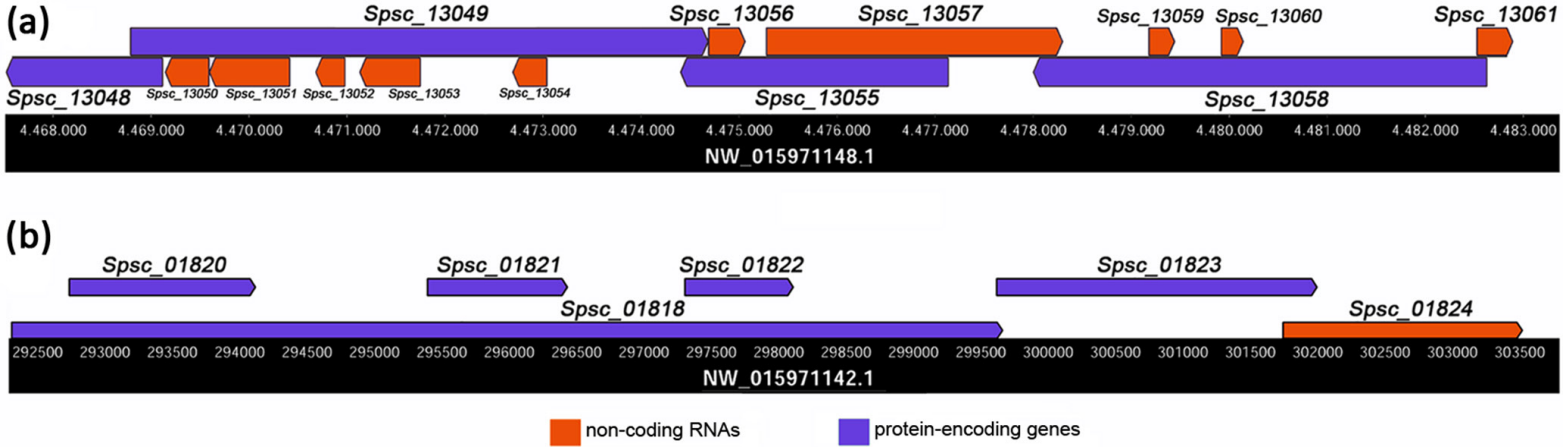
Snapshots of the newly annotated genomic regions of *S. schenckii*. (a) Uninterrupted chromosome region containing multiple sense–antisense transcripts assorted in a complex configuration. The image shows the largest gene group detected containing 14 different-strand overlapping genes. (b) Uninterrupted chromosome region containing one of the three largest gene groups involving six same-strand overlapping genes.

In this study, we performed strand-specific RNA sequencing to retain the strand origin of the transcripts; thus, allowing us to predict sense RNAs and asRNAs (Fig. 1), identifying exactly the overlapping regions of transcription and estimating accurately the expression levels of sense and antisense genes. Using this approach, we identified a number of genomic regions, generically known as ‘transcriptionally active regions’ (TARs) or ‘transcribed fragments’ (transfrags) [38], that contain a pair (or more) of distinct genes mapping to opposite DNA strands (Table S1). Technically, two genes that reside on opposite genomic strands within the same locus and share sequence overlap (at least 1 nucleotide) can be defined as a cis-SAS gene pair [39] that can be structurally classified into four different types (divergent, convergent, embedded and fully overlapping) based on the overlapping ends of the sense–antisense transcripts and on their reciprocal direction of transcription [40] (Fig. 2c). In our study, the extent of the overlapping region between sense transcripts and complementary asRNAs was highly variable, ranging from 1 to 22 569 nucleotides (Table S1). Of the 7820 overlapping regions detected, 2066 held only one pair of complementary transcripts (called paired sense–antisense overlapping genes) (Table 3, Fig. 2a) produced from opposite DNA strands (Table S1). Most of them (603/2066; ~29.2 %) showed a convergent configuration (3′-ends overlap), followed by embedded (591/2066;~28.6 %), fully overlapping (578/2066;~28.0 %) and divergent conformations (5′-ends overlap) (294/2066;~14.2 %) (Fig. 2c). The remaining genomic regions were assorted in different and more complex configurations containing multiple sense–antisense transcripts or overlapping gene clusters (Table 3). Some examples are depicted in Fig. 4. However, for a more in-depth structural exploration of *S. schenckii* gene models, it is possible to inspect the new genome annotation (v2) at http://sporothrixgenomedatabase.unime.it.

**Table 3.** List of gene groups found among different- and same-strand overlapping genes detected in this study

Gene group (no. of genes within each group)	No. of each gene group found in *S. schenckii*	Total no. of genes detected within each gene group	Frequency (%)*
**Gene groups obtained from different-strand overlapping genes**			
Paired (2)	2066	4132	35.2
Triple (3)	885	2655	22.6
Quadruple (4)	433	1732	14.8
Quintuple (5)	236	1180	10.1
Sextuple (6)	114	684	5.8
Septuple (7)	72	504	4.3
Octuple (8)	43	344	2.9
Nonuple (9)	25	225	1.9
Decuple (10)	9	90	0.8
Undecuple (11)	3	33	0.3
Duodecuple (12)	7	84	0.7
Tredecuple (13)	4	52	0.4
Quattuordecuple (14)	1	14	0.1
**Gene groups obtained from same-strand overlapping genes**			
Paired (2)	781	1562	73.6
Triple (3)	127	381	18.0
Quadruple (4)	35	140	6.6
Quintuple (5)	5	20	0.9
Sextuple (6)	3	18	0.8

*Percentage of genes of each group for total different-strand overlapping genes (11 729) and for total same-strand overlapping genes (2121).

Examining the various cis-SAS gene pairs occurring in the *S. schenckii* genome, we found that they contain both protein-encoding genes and non-coding RNA (ncRNA) genes, albeit the most common model includes one coding strand and one non-coding strand in each pair (Table S1). However, the bi-directional transcription of a generic cis-SAS gene pair can lead to both coding and/or non-coding asRNAs that overlap, partially or completely, with other coding or non-coding sequences; thus, highlighting the extremely sophisticated organization of the *S. schenckii* genome. In fact, up to ~39 % (3034/7820) of sense–antisense overlapping regions detected produced protein-encoding asRNAs (transfrags with significant alignment to proteins deposited in the UniProt database) with a partial (or complete) overlap with the corresponding sense protein-encoding sequence (Table S1). Interestingly, a total of 243 of these overlapping regions showed a fully overlapping configuration (Fig. 2c), indicating that they are entirely transcribed in both directions and that two different mRNAs are produced (Table S1). As a consequence, we found that ~4 % (486/11 217) of the *S. schenckii* protein-encoding genes are bi-directionally transcribed with opposite polarity. The remaining overlapping regions (4786) encoded a number of transcripts that were predicted to be potential antisense ncRNAs (ancRNAs) by the cpc2 program. Over 87 % of these regions (4171/4786) transcribed ncRNAs (3544 non-redundant non-coding transcripts) (Table S1) that overlap neighbouring protein-encoding genes, whereas ~13 % (615/4786) produced 1211 unique (non-redundant) ncRNAs that overlap each other (Table S1).

### Overlapping gene groups in the *S. schenckii* genome

To investigate the occurrence of overlapping gene types in *S. schenckii*, we used 11 217 protein-encoding and 5929 lncRNA genes annotated in this study by using strand-specific RNA-seq data (Table 1). Most of the overlapping genes detected (11 729) belong to the different-strand overlapping type (Fig. 2a, Table 3) as their transcripts map within 7820 overlapping regions that can be transcribed in both sense and antisense directions. By estimating the gene density in each of these uninterrupted overlapping regions, we found different clusters of overlapping cis-SAS genes ranging from 2 to 14 genes (Table 3). The paired sense–antisense overlapping genes constituted the most common type (4132/11 729; 35.2 %), followed by the triple (2655/11 729; 22.6 %), quadruple (1732/11 729; 14.8 %), quintuple (1180/11 729; 10.1 %), sextuple (684/11 729; 5.8 %) and septuple overlapping genes (504/11 729; 4.3 %), and other minor groups containing from 8 to 14 gene clusters that together make up ~7 % of all overlapping sense–antisense gene groups detected (Table 3). The chromosome region reported in Fig. 4(a) contains the unique largest overlapping gene group (14 genes involved) found in the *S. schenckii* genome. Based on these data, we estimated that over 68 % (11 729/17 146) of the *S. schenckii* genes are found to overlap and many of them are tightly compacted into genomic regions in which transcription may occur from either strand of the DNA molecule. However, we also identified a total of 1171 genomic regions containing 2121 same-strand overlapping genes (Tables 3 and S1), although it should be noted that our approach for classifying the different gene overlaps introduces the possibility of redundancy, as some transcripts may be represented more than once in the different categories of overlap (the same gene may share both different- and same-strand overlaps). Consequently, we estimated that approximately ~71 % (12 111 non-redundant genes) of the 17 146 S. *schenckii* protein-encoding/lncRNA genes were found to overlap in some way.

Similarly to different-strand, for same-strand overlapping genes, the paired configuration was the most common type (1562/2121; 73.6 %), although the same-strand overlapping genes were grouped into only five groups (Table 3). The triple and quadruple overlapping gene groups accounted for 18 and 6.6%, respectively, whereas the quintuple and sextuple overlapping gene groups were the least abundant among all the same-strand overlapping genes detected (<1 % each; Table 3). The chromosome region displayed in Fig. 4(b) shows the organization of one of the three gene clusters found involving six same-strand overlapping genes.

### Differential gene expression analysis

**Fig. 5.**
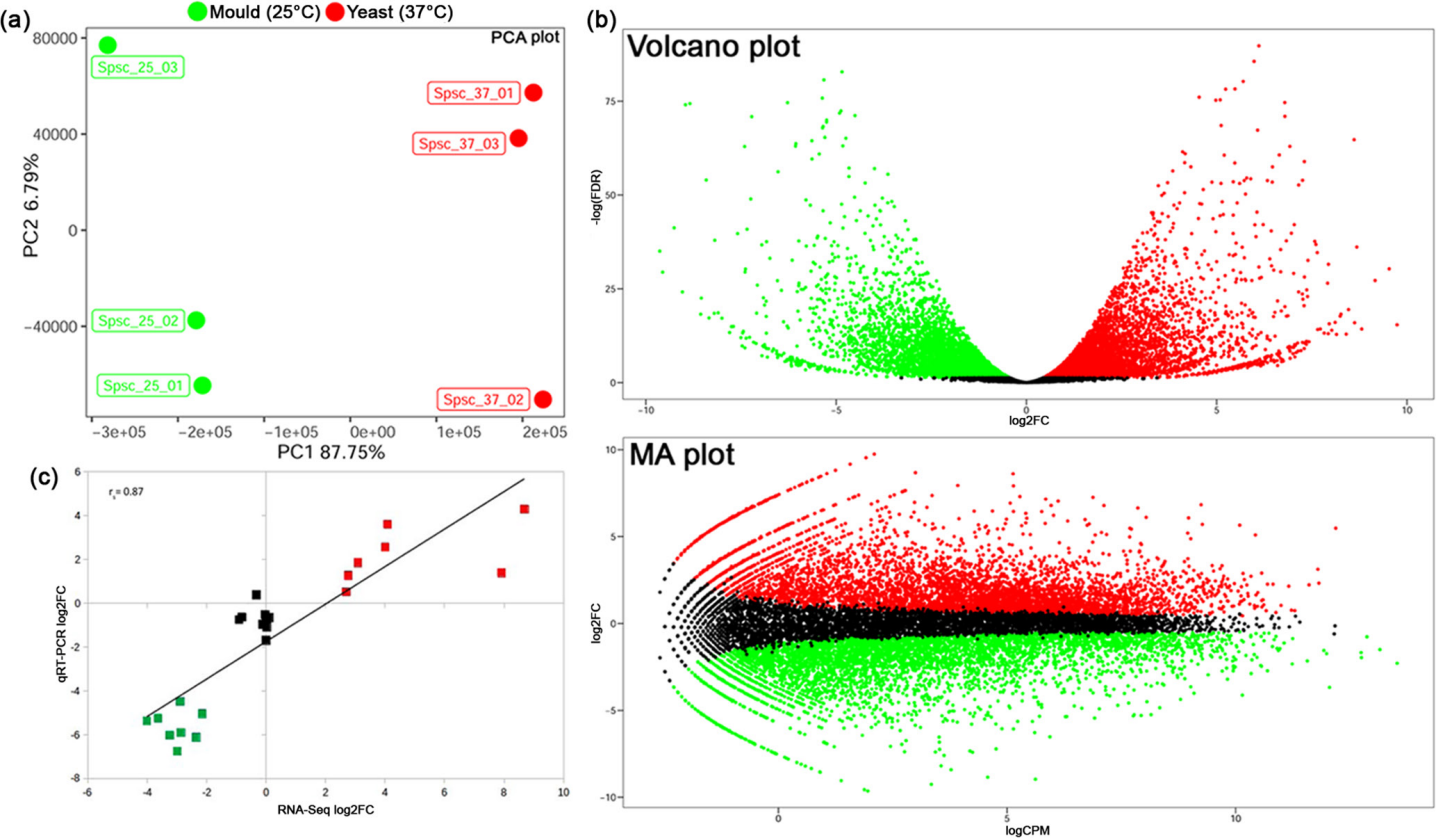
Sample variability, validation of RNA-seq data by qRT-PCR and differential gene expression between yeast and mould samples of *S. schenckii* examined in this study. (a) Principal component analysis (PCA) plot showing transcriptional profiles of *S. schenckii* cells grown at 25 °C (SSC_1–3 green) and 37 °C (SSC_4–6 red). The two groups are visually well-separated by the PC1 dimension and there is only a minor variation among the biological replicates as evidenced by the tenfold smaller scale for dimension PC2 compared to PC1. (b) Volcano plot and MA plot displaying differential gene expression between yeast and mould forms of *S. schenckii*. Significantly up-regulated and down-regulated genes in yeast cells compared with mould cells are indicated by red and green dots, respectively. Black dots indicate genes with no significant differences in their expression levels between the two tested conditions. (c) Scatter plot showing the correlation between RNA-seq and qRT-PCR expression data for 24 selected genes. The log_2_FC value is displayed for both RNA-seq data, as obtained from edgeR software, and qRT-PCR results, obtained from log_2_(2^−ΔΔ*C*_t_^). The Spearman’s correlation coefficient r_s_ is displayed. Squares denote up-regulated (red), down-regulated (green) and not regulated (black) genes.

The main goal of this study was the characterization of the transcriptomic differences between yeast and mycelial forms of *S. schenckii*. The principal component analysis clearly separated the yeast and mycelial samples, along the first axis, into two well-defined groups (Fig. 5a), indicating that the observed variation (87.75 %), measured in terms of gene expression, was due to dimorphism. There was only a small variation (6.79 %) between biological replicates within each morphotype (Fig. 5a).

Using our new genome annotation, global gene expression analysis revealed a catalogue of 12 104 and 12 911 genes expressed (FPKM >1) in the yeast-phase and mould-form, respectively. Of these, 10 443 genes were found to be expressed in both morphotypes (Table S2), while 1661 and 2468 were yeast-specific and mould-specific genes, respectively (Table S2). However, in total, 8795 genes were found to be differentially regulated among yeast and mould forms of *S. schenckii* when an absolute log_2_FC value ≥1 and an FDR threshold of 0.05 were used (Fig. 5b, Table S3). Collectively, these genes represent ~51 % (8795/17 307) of all annotated *S. schenckii* genes and more than half of them (4494 genes) were found to be up-regulated in the yeast-form (Fig. 5b, Table S3).

qRT-PCR results confirmed the RNA-seq expression patterns observed for the selected differentially up-/down-regulated and unregulated genes. Comparison of edgeR estimated expression data with those determined by qRT-PCR showed a significant positive correlation (r_s_=0.87) between the two measures of gene expression levels (Fig. 5c).

RNA-seq analysis also revealed a large amount of variation in ancRNA expression. In fact, among the 4171 ancRNA overlapping protein-encoding loci, 2236 unique ancRNAs were found to be statistically differentially expressed (absolute log_2_FC ≥1; FDR ≤0.05) between the two morphological types examined (Table S4). Interestingly, 1117 protein-encoding genes that were not differentially expressed (FDR>0.05) among yeast and mould forms showed their antisense transcript partners to be differentially regulated in the two conditions (Table S4).

### GO term enrichment analysis of DE genes

GO enrichment analysis was performed to identify and classify the potential molecular function exerted by DE genes, including the different biological processes in which they are involved and the cellular locations where these occur (Table S5). Based on GO analysis of 8795 DE genes, we found that 5624 (~64 %) were categorized in a total of 2629 functional groups or classes (GO terms) (Table S5). However, when data were filtered using an FDR cut-off <0.05, we detected a total of 1318 GO terms in the entire data set (Table S5). More specifically, 1030 genes were classified into 542 functional groups within the main domain of biological process, 1455 genes into 525 functional groups within molecular function, and 835 genes were categorized into 251 classes within the cellular component domain (Table S5).

Next, we performed a GO enrichment analysis for genes that were specifically up-regulated (4494 genes) and down-regulated (4301 genes) between the two morphological stages of *S. schenckii* (yeast versus mould) (Table S3). The results are reported in Table S6. After FDR filtering (cut-off <0.05), up-regulated genes were categorized in a total of 664 functional groups, of which 270 belonged to biological process, 270 to molecular function, and 124 to the cellular component domain.

**Fig. 6.**
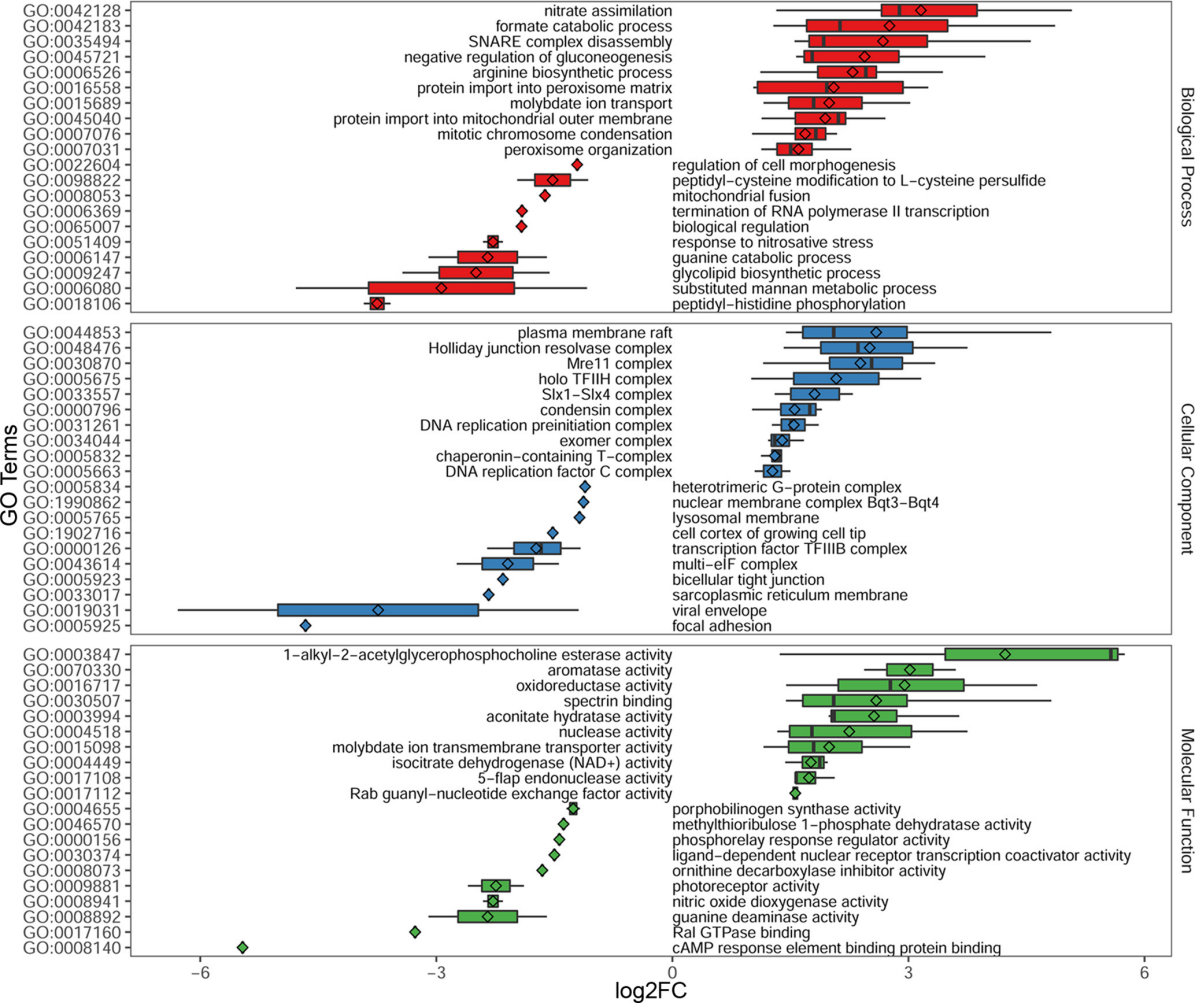
Box plot representation of the top 10 enriched GO terms of DE genes for each ontology category (biological process, cellular component and molecular function). The rhombuses inside each box indicate the mean of the log_2_FC values of genes included in the respective GO term.

Regarding down-regulated genes, a total of 321 GO terms were found at the FDR <0.05 threshold. Of these, 136 were within the domain of biological processes, 132 within molecular function and 53 in the cellular component category. Fig. 6 displays the top 10 GO terms assigned to up- and down-regulated genes in the three main ontologies.

## Discussion

In this study, we generated high-quality RNA-seq data that were used for obtaining a complete view of the genes expressed by the dimorphic fungus *S. schenckii* under hyphae- and yeast-inducing conditions. This comprehensive transcriptome dataset can be queried and explored at http://sporothrixgenomedatabase.unime.it, where RNA-seq data, including the new genome annotation and gene expression profiles, can be also freely downloaded for further investigation. A point of special interest in this study is the detailed genomic description of thousands (5929) of lncRNA loci that make up over 34 % of the total genes occurring in the *S. schenckii* genome. A similar number has been reported recently in other fungal genomes [41, 42], but their abundance may vary widely among different species with different genome sizes [43]. However, the real occurrence of lncRNAs in fungi is, currently, likely underestimated, because the detection of these regulatory elements largely depends on the type of experimental condition examined and on the sequenced transcripts which, as in our study, are often poly(A) selected; thus, precluding the possibility of capturing the non-polyadenylated lncRNAs [44]. Anyhow, by uncovering the expansive landscape of lncRNAs associated with the yeast and/or mould forms of *S. schenckii*, we provide the scientific community with a powerful starting point to begin investigating their biological relevance.

Undoubtedly, an interesting aspect of lncRNA biogenesis in *S. schenckii* is that most lncRNAs (~77 %) are transcribed from overlapping loci (cis-SAS gene pairs) and only a small fraction originates from intergenic or other genomic regions (~23 %). These results are in contrast with the very few findings dealing with lncRNAs in other fungi [43, 45, 46], in which intergenic regions appear to be the main sources of these RNA molecules. However, our data are in line with the results of two recent studies in *Schizosaccharomyces pombe* [41] and *Nosema ceranae* [47], where most of the discovered lncRNAs were transcribed as natural antisense transcripts (NATs). NATs are RNA molecules that originate from opposite DNA strands of the same genomic locus (cis-NAT) or unlinked loci (trans-NAT), and can be generically defined as coding RNAs or ncRNAs that are complementary to, and overlap with, either protein-encoding or non-coding transcripts [48]. Many cis-NATs encode lncRNAs that have been reported as important regulators of gene expression in a wide range of ascomycete and basidiomycete fungi [40]. In *S. schenckii*, we found a total of 11 729 cis-SAS genes which represent, to our knowledge, the largest number of overlapping sense–antisense genes reported in fungi to date. However, through comprehensive transcriptomic studies, thousands of NATs have been detected in other fungi also, including *Magnaporthe oryzae* (4215), *Schizosaccharomyces pombe* (4384) and *Schizophyllum commune* (5635), but their functional roles remain to be investigated [40]. Interestingly, in our study, we observed different clusters of overlapping cis-SAS genes ranging from 2 (paired overlapping genes) to 14 genes. Similar multigene-transcript overlapping regions have also recently been described in the human genome, where about 72 % of the overlapping protein-encoding genes showed a paired configuration, followed by more complex overlapping groups until reaching 22 overlapping genes in a protocadherin gene cluster on chromosome 5 [49]. Consequently, in terms of gene overlap, our data suggest that the physical chromosomal arrangement of the *S. schenckii* genes follows, to some extent, the pattern observed in other eukaryotic genomes where the different-strand overlapping type seems to be the most commonly adopted gene model [30]. In this regard, it is worth noting that this contrasts with what has been observed in bacteria, where the genes that overlap on the same-strand are by far the most abundant [50]. This different gene disposition could probably be linked to the presence of a high number of antisense lncRNAs occurring in eukaryotes compared to prokaryotic genomes in which this class of RNA molecules is rare [51]. Furthermore, the existence of overlapping genes in *S. schenckii* and other eukaryotic genomes should be carefully considered in future gene manipulation and knockout studies, because possible polar effects could appear by manipulation of one overlapping locus, especially those in which both DNA strands encode different proteins.

Another important aspect of our study concerns the genome-wide analysis of gene expression, which emphasizes the extraordinary complexity of the molecular pathways that control either genesis and maintenance of yeast and hyphae cell morphologies, which could be governed by the expression of different lncRNA molecules and by a number of still uncharacterized protein-encoding genes (Table S3). Interestingly, GO enrichment analysis using DE genes revealed an overall up-regulation of genes involved in nitrate assimilation and arginine biosynthesis (Fig. 6). In fungi, nitric oxide and l-arginine have been implicated in several cellular processes, including the regulation of morphogenesis and reproduction [52, 53]. In particular, in *Coniothyrium minitans* and *M. oryzae*, l-arginine plays an important role in the asexual developmental process and is essential for conidiation [52, 54]. In fact, in *Coniothyrium minitans*, the disruption of the carbamoyl-phosphate synthase (CSP1)-encoding gene (a key gene in the biosynthesis of l-arginine) yielded a conidiation deficiency mutant that could only grow hyphae in culture [52]. Disruption of the gene ortholog in *M. oryzae* (*CPA2* gene) also impairs conidiogenesis and its transcript levels were significantly lower in mycelial growth compared to the conidial stage [54]. Similarly, we found that the expression of the *S. schenckii* carbamoyl-phosphate synthase gene (ORF Spsc_00280) was down-regulated in the mould-form compared to the yeast-form, suggesting a possible involvement of the amino acid biosynthetic pathways in hyphae-to-yeast transition, an assumption supported by a recent work exploring the crucial role of metabolic pathways in *Candida albicans* morphogenesis [55].

In *S. schenckii*, few studies have been conducted to identify key genes involved in triggering and maintaining the dimorphic changes. One gene (*DRK1*), encoding a hybrid histidine kinase (HHK), has been described as a master regulator of the hyphae-to-yeast transition and virulence in several dimorphic fungi, including *S. schenckii* [56, 57]. The expression of this gene was reported to be up-regulated in the yeast-phase compared to the mould-phase [56, 57] but, in our study, we found it (ORF Spsc_00605) to be not differentially expressed between mould and yeast forms of *S. schenckii* (Table S3). This result was also confirmed by using a qRT-PCR assay (Table S1). In our opinion, this discrepancy in *DRK1* expression levels could be explained by the different experimental conditions employed for obtaining the yeast and mould phases by various studies. In fact, in our study, both morphologies were obtained by growing the fungus in YPD medium up to 6 days, while in previous studies [56, 57], the mould and yeast forms were obtained by using different incubation times (≤4 days) and culture media: Sabouraud liquid medium for mould growth and brain heart infusion broth for the yeast development. However, based on our data, it is possible that in *S. schenckii*, after induction of the yeast-phase, the *DRK1* gene expression level decreases and other factors contribute to maintaining the yeast form. Interestingly, in this study, we also found an additional HHK encoded by the ORF Spsc_09813. This new HHK was found to be differentially expressed between the two examined *S. schenckii* morphologies, it was up-regulated in the yeast-form. Bioinformatics analysis of the Spsc_09813 transcript revealed that it encodes a protein carrying all structural domains specific for fungal HHKs [58]: (i) a N-terminus Per-Arnt-Sim (PAS_9) sensing domain; followed by (ii) a central region composed of a histidine kinase (dimerization/phosphoacceptor) domain (HisKA) and a cognate histidine kinase-like ATPase catalytic domain (HATPase_c); and (iii) a conserved C-terminus region referred to as receiver domain [58]. However, the molecular mechanisms leading to dimorphic switching in fungi are very complex and depend on the expression of many genes. In fact, this phenomenon can be induced by different biophysical stimuli that operate through specific or cross-talk molecular signalling pathways [59], which, in turn, activate specific downstream proteins whose transcription can also be finely regulated by ncRNAs. This latter assumption has been recently proven by a study in *Cryptococcus* in which a specific lncRNA, encoded by the *RZE1* gene, controls the yeast-to-hypha transition in this fungus [60]. Moreover, many other lncRNAs seem to have important roles in fungal development and stress response [61, 62]; therefore, the functional characterization of the thousands of lncRNAs differentially expressed in *S. schenckii* (Table S3) could give new insight on the capacity of these non-coding elements in regulating morphogenesis in this species also. The transcriptomic data reported in this study will not only serve as a valuable reference for investigation of genes involved in the dimorphic transition and other biological processes, but also expand our knowledge beyond what has been learnt through recent genomic studies of different *Sporothrix* species [6, 13, 63, 64].
